# Impact of Pharmacogenetic Testing on Clozapine Treatment Efficacy in Patients with Treatment-Resistant Schizophrenia

**DOI:** 10.3390/biomedicines12030597

**Published:** 2024-03-07

**Authors:** Estela Sangüesa, Emilio Fernández-Egea, Julia Concha, Cristina B. García, María Pilar Ribate

**Affiliations:** 1 Facultad de Ciencias de la Salud, Universidad San Jorge, Villanueva de Gállego, E-50830 Zaragoza, Spain; esanguesa@usj.es (E.S.); jconcha@usj.es (J.C.); mpribate@usj.es (M.P.R.); 2 Cambridgeshire and Peterborough NHS Foundation Trust, Cambridge CB21 5EF, UK; ef280@cam.ac.uk; 3 Department of Psychiatry, Behavioural and Clinical Neuroscience Institute, University of Cambridge, Cambridge CB2 0SZ, UK

**Keywords:** antipsychotics, pharmacodynamics, drug response, pharmacogenetic variants, genetic testing, treatment-resistant schizophrenia

## Abstract

Managing schizophrenia with clozapine poses a significant challenge due to prevalent therapeutic failures. The increasing interest in personalized medicine underscores the importance of integrating pharmacogenetic information for effective pharmacotherapeutic monitoring in patients. The objective of this study was to explore the correlation between *DRD2*, *HTR2A*, *SLC6A4*, *CYP1A2*, and *ABCB1* polymorphisms and clozapine response in 100 patients with Treatment-Resistant Schizophrenia. Different scales such as the Positive and Negative Syndrome Scale (PANSS), the Warwick-Edinburgh Mental Wellbeing Scale (SWEMWBS), the Global Assessment of Functioning Scale (GAF), the Brief Negative Symptom Scale (BNSS), and pharmacokinetic parameters were used to analyse the efficacy of the treatment. Patients who exclusively responded to clozapine compared to the patients with augmentation strategies exhibited distinctive features, such as lower doses, plasma levels, and presented less-pronounced symptomatology. Genetic associations were explored, highlighting *SLC6A4, HTR2A*, and the *1F/*1F polymorphism for the *CYP1A2* gene.

## 1. Introduction

Clozapine (CLZ) is an atypical antipsychotic that has been found to be effective in treating resistant schizophrenia (TRS), yet it is underutilized due to concerns about its potential side effects and the need for close monitoring. CLZ acts on both positive and negative symptoms as well as on cognitive dysfunction and it has higher efficacy and tolerability compared with other antipsychotics [1]. However, about 30–50% of patients receiving CLZ do not respond adequately or develop severe side effects, such as potentially fatal agranulocytosis [2,3]. The causes behind the lack of response or the occurrence of certain side effects are difficult to discern because the CLZ mechanism of action is complex and still not entirely clear [4]. It possibly depends on multifactorial processes. Therefore, several studies have been performed in order to identify predictors of CLZ response, such as clinical factors (gender, age, baseline body weight, baseline body mass index), therapeutic drug monitoring (TDM) of CLZ and its metabolites, or single nucleotide polymorphisms (SNPs) [5,6,7,8]. 

Genetic polymorphisms may explain the treatment failure in some patients because they can alter the expression and activity of metabolizing enzymes, transporters or the structure of drug targets, causing interindividual differences in pharmacokinetics and pharmacodynamics [9]. To date, there are no treatment recommendations based on any pharmacodynamic-related gene variants available for antipsychotics. But, CLZ interacts with various neurotransmitter receptors, such as serotonin 5-HT_2_A and dopamine receptors [10,11], so a genetic susceptibility between these pharmacodynamics variables may contribute to the lack of response. 

CLZ is characterized by its high selectivity for the mesolimbic system, with a preference for D_2_ and D_4_ receptors, and a weak partial binding to the D_2_ receptor (DRD_2_) in the nigrostriatal pathway [12]. Given the fast-off-D_2_ phenomenon of CLZ to DRD_2_, polymorphisms and variants of the gene encoding this receptor (*DRD2*) have been studied to establish the potential impact it may have on the efficacy of treatment [13,14]. The Taq1A (rs1800497) polymorphism has been observed to influence D_2_ receptor expression, with the A1 allele associated with reduced D_2_ receptor density in the caudate and putamen [15,16]. For that, A1/A1 carriers showed a significantly better response using different scales according to psychiatric symptoms in comparison to noncarriers in different antipsychotic treatments [17,18]. 

The 5-HT_2_A receptor is a post-synaptic G protein-coupled receptor with which CLZ has high affinity [19]. The T allele of rs6313 polymorphism of the *HTR2A* gene has been associated with good response to CLZ [20,21], although controversial results have also been demonstrated [22,23]. A recent study confirms the association with *HTR2A* rs6313 T allele carriers showing generally better improvement than non-carriers in risperidone and olanzapine treatment [24]. The *HTR2A* gene also contains a SNP (rs6314), implying a missense substitution His/Tyr at amino acid 452 in the C-terminal region of the receptor [25]. This missense substitution affects the function of the receptor with lesser efficacy of the drug and has been associated with worse response to CLZ [26,27,28]. Arranz et al. determined the clinical response using the Global Assessment of Functioning Scale (GAF) and a 20-point improvement in the scale after CLZ treatment was considered as cut-off for response [26,27,28]. Masellis et al. evaluated the treatment response based on a reduction of >20% in the Brief Psychiatric Rating Scale (BPRS) score from a baseline score at enrolment into the study [28].

The *SLC6A4* gene, which encodes the serotonin reuptake transporter protein, contains a polymorphism in its promoter region (5-HTTLPR) that has been extensively studied. It can have two variants whose naming depends on their size: the S (short) allele and the L (long) allele, resulting from an indel. Another polymorphism in this gene (rs25531) provides additional information with two other alleles related to the previous one, adenine (A)/guanine (G). These variations have been investigated in terms of their relationship with symptoms and CLZ response based on a 30% reduction of the scores of BPRS for appropriate response with drug adjustment up to 900 mg/day [29]. 

Another related biomarker that could help to explore interindividual variability and drug failure is the drug plasma concentration. Thus, TDM is recommended for CLZ follow-up. The suggested therapeutic reference range for a therapeutically effective dose in the treatment of adult patients with schizophrenia is 350–600 ng/mL for CLZ and 100–600 ng/mL for its metabolite [30]. However, TDM has limits in identifying the origin of the therapeutic failure without being able to explain the cause of some abnormal results in plasma levels or recurrent drug failures [31]. In these cases, pharmacogenetic analysis of the drug’s pharmacokinetic variables could help to explain the abnormal results obtained. CLZ is mainly metabolized by CYP1A2. The genotype *1F/*1F for the *CYP1A2* rs762551 gene results in ultra-rapid metabolism, impacting the pharmacokinetics of CLZ. On the other side, P-glycoprotein (P-gp) functions as a membrane transporter protein tasked with removing molecules from the central nervous system into the bloodstream, as well as from the bloodstream into the gastrointestinal tract, bile, and urine [32]. Consequently, elevated CYP (CYP1A2*1F rs762551) or P-gp activity (*ABCB1* rs1045642 and rs2032582) could decrease the CLZ blood concentration, which may lead to a lack of therapeutic effect [8,33,34]. Genetic polymorphisms of the genes encoding these enzymes and transporters represent a factor in interindividual variability in drug response. Genetic predictors of CLZ response could be helpful in the future in offering personalized therapeutic strategies for these patients.

Therefore, the aim of this study was to investigate the potential association of selected polymorphisms, located in the *DRD2*, *HTR2A*, *SLC6A4*, *CYP1A2,* and *ABCB1* genes, with CLZ response in TRS, by using different scales such as the Positive and Negative Syndrome Scale (PANSS), the Warwick-Edinburgh Mental Wellbeing Scale (SWEMWBS), the Global Assessment of Functioning Scale (GAF), the Brief Negative Symptom Scale (BNSS), and pharmacokinetic parameters.

## 2. Materials and Methods

### 2.1. Participants and Design

This longitudinal observation study consisted of 100 participants from the Cambridgeshire and Peterborough NHS Foundation Trust (CPFT) covering information from 24 August 2012 to 31 December 2022. The Clinical and Research Database (CRD) for Persistent Schizophrenia was used under NHS Research Ethics Committee (REC) approvals (ref. 13/EE/0121; 18/EE/0239). This research database contains anonymous information about routine clinical data from the CPFT Clozapine Clinic [35]. Extracted data maintained patient anonymity by removing all identifiable data. All clinical assessments in the CRD were performed by an experienced psychiatrist (EFE) or self-rated by the patient during routine clinical appointments.

The patient eligibility criteria were as follows: adult patients (age > 18 years) with non-affective psychosis and on CLZ treatment. Only those capable of giving their consent were included, which included those who lived independently in the community.

### 2.2. Routine Clinical Assessments

All annual care plan assessments (CPAs) in the electronic record include relevant sociodemographic data (age, gender, age of illness onset, date CLZ start), concurrent use of other psychotropic drugs (including inductor or inhibitors of CLZ metabolism), current smoking habit (average number of cigarettes per day), and latest CLZ and norclozapine (NCLZ: active metabolite) plasma levels results (including date of test). Relevant psychopathology scales included in the CRD include the Global Assessment of Functioning Scale (GAF), the Short Warwick-Edinburgh Mental Wellbeing Scale (SWEMWBS), the positive subscale of the Positive and Negative Symptoms Scale (PANSS-positive) [36], and the Brief Negative Symptom Scale (BNSS). Negative symptoms were assessed using the 13-item BNSS. We utilized the two primary clinical factors, the Motivation and Pleasure Factor (sum of items 1–3 and 5–8) and the Expression Factor (sum of items 9–13) [37].

According to the pharmacotherapeutic algorithm for schizophrenia, a patient who has shown an inadequate clinical response after two attempts with appropriate antipsychotics is recommended to receive CLZ as monotherapy. However, if it is observed that the patient has not exhibited an adequate response to CLZ treatment, strategies for augmentation are proposed, involving the combination of two antipsychotics with different therapeutic targets, or the use of long-acting injectable antipsychotics in cases of low patient adherence or patient preference. This subgroup of TRS is known as Ultra Treatment Resistant Schizophrenia (UTRS) [38]. Following these criteria, patients were categorized as CLZ responders (CLZ-TRS) if they met the first condition, and as CLZ ultra treatment resistance (CLZ-UTRS) if they required augmentation strategies.

### 2.3. Determination of Plasma Levels

As per internal protocol, the CPFT Clozapine Clinic included the analysis of CLZ and NCLZ plasma levels routinary. To obtain CLZ trough concentrations, it was ensured that the patient underwent an overnight fast and the last dose was taken 12 h prior to blood collection. All patients were on stable doses of CLZ with no change in the previous 28 days. A two mL blood sample was taken by venipuncture within EDTA tubes. CLZ and NCLZ plasma levels (mg/L) were measured at the Toxicology Department, King’s College Hospital, London, UK by Liquid Chromatography-Tandem masses (LC-MS/MS) and then converted in ng/mL.

### 2.4. Pharmacogenetic Analysis

One hundred patients also consented to participate in the ethics-approved “Genetics of common clozapine-induced side effects” study (REC 18/NW/0581). From each patient, a 0.5 mL aliquot of the peripheral blood sample was stored in FTA^TM^ cards for the purpose of DNA extraction and subsequent genotyping. Genetic information analysis was conducted at San Jorge University in Zaragoza. The study analysed CLZ drug response associated with 8 polymorphisms in the drug molecular targets and main metabolizing enzymes genes in patients with TRS (Table 1).

Genotyping of *CYP1A2* (rs7625521/*1F)*,* drug transporter P-gp/*ABCB1* (rs2032582), and *HTR2A* (rs6313 and rs6314) was performed by Real Time PCR using rhAmp^®^ SNP Assay probes (Integrated DNA Technology (IDT^®^), Coralville, IA, USA). Genotyping of *DRD2* (rs1800497) and *ABCB1* (rs1045642) was performed by Real Time PCR using TaqMan^®^ SNP assay (Applied Biosystems, Carlsbad, CA, USA). Genetic variants of the *SLC6A4* gene, the 5-HTTLPR polymorphism rs4795541 and rs25531, were genotyped using MJ Mini Thermal Cycler (Bio-Rad, Hercules, CA, USA) following PCR-RFLP adapted from previous studies [39]. Amplified PCR products were subjected to restriction digestion with appropriate enzyme *(MspI*) for *SLC6A4* rs25531 polymorphism.

### 2.5. Statistical Analysis

The statistical analysis was computed using SPSS software for Windows 28.0.0.1 (IBM Corporation, Armonk, NY, USA). The categorical variables were reported as frequencies and percentages. The continuous variables were expressed by mean, median, standard deviation, and range according to normal distribution. Normal distribution of these variables was evaluated according to the Kolmogorov–Smirnov and Shapiro–Wilk tests. CLZ metabolic capacity was defined as the dose-adjusted plasma concentration (concentration/daily dose ratio, C/D). The dose-adjusted plasma concentrations (concentration/daily dose ratio) for CLZ (C/D-CLZ) and NCLZ (C/D-NCLZ) were calculated in ng/mL per mg of daily dose. Also, total C/D was calculated by dividing the sum of CLZ and NCLZ by CLZ daily dose. The differences between these two independent groups were analysed by Student’s *t*-test. The differences between three or more independent groups were determined by ANOVA test. A *p*-value ≤ 0.05 was assumed to be statistically significant.

Bivariate correlation and multiple linear regressions were also employed to analyse the association between CLZ pharmacokinetic parameters and demographical data and concomitant medication.

A binary logistic regression was performed using the “Enter” method, with the level of drug resistance (CLZ-TRS vs. CLZ-URS) designated as the dependent variable and the genotypes of the studied polymorphisms as independent categorical variables. The threshold for statistical significance was established at (two-tailed) *p* ≤ 0.05.

## 3. Results

All the demographic (age, gender, smoking) and clinical data (pharmacokinetic parameters and scales) are shown in Table 2.

An extensive examination by bivariate correlation and multiple lineal regression of the non-genetic factors employed in this study did not reveal significant associations between sociodemographic (age and gender), concomitant medications (inductors and inhibitors of CLZ), and any of the CLZ pharmacokinetic parameters (plasma levels and C/D ratios of CLZ and NCLZ).

One hundred patients with schizophrenia receiving CLZ were divided into two groups based on the need for a second antipsychotic medication after receiving CLZ. Of the total 100 patients, n = 49 were undergoing treatment only with CLZ as an antipsychotic (CLZ-TRS), and n = 51 patients were on treatment with CLZ and one or more other antipsychotics from the first or second generation (CLZ-UTRS), including aripiprazole (41%), risperidone (1%), sulpiride (8%), amisulpride (9%), or haloperidol (1%).

In the group exclusively taking CLZ, the average daily tobacco consumption was 7.89 cigarettes (SD = 1.59) (n = 28 (57.1%) non-smokers vs. n = 21 (42.9%) smokers). Gender distribution showed 41 males (83.7%) and 8 females (16.3%), with a mean age of 51.36 years (SD = 1.63). In contrast, for individuals on CLZ-UTRS, the average daily tobacco intake was 5.14 cigarettes (SD = 1.37) (n = 31 (60.8% non-smokers vs. n = 20 (39.2%) smokers). The gender composition indicated 45 males (88.2%) and 6 females (11.8%), with an average age of 49.19 years (SD = 1.51).

The patients who responded to only CLZ compared to those requiring augmentation strategies were treated with lower doses of CLZ (*p* = 0.033), exhibited lower plasma levels of CLZ and NCLZ (*p* < 0.001), and showed higher Wellbeing Score values (*p* = 0.003). They presented less pronounced symptomatology, both in terms of negative and psychotic symptoms (*p* ≤ 0.05) (Table 3).

The logistic regression model utilized was not significant in predicting CLZ-UTRS (*p* > 0.05), and none of the genotypes from the analysed genes significantly predicted membership in the CLZ-UTRS group.

### 3.1. Associations of Gene Polymorphisms Related with Pharmacodynamics and CLZ Response

All the polymorphisms of *DRD2*, *HTR2A*, and *SLC6A4* were analysed in relation to pharmacokinetic parameters (dose, plasma levels, and relation concentration versus dose) and different scales. According to the pharmacokinetic parameters only the 5-HTTLPR (rs4795541) polymorphism showed an association. Our analysis showed that only when comparing the mean plasma levels of CLZ and NCLZ for 5-HTTLPR S allele carriers versus L allele carriers, a significant difference was obtained (370 vs. 445 ng/mL CLZ plasma levels *p* = 0.008; 232 vs. 263 ng/mL NCLZ plasma levels *p* = 0.049). The S allele carriers showed the lowest CLZ and NCLZ plasma levels versus L allele carriers. When patients were divided according to genotypes and haplotypes for *SLC6A4* gene, no differences between groups were found.

When investigating whether the psychopathology scales were different in carriers of *HTR2A* rs6314 allele, we observed several significant associations with the scores on PANSS-positive (*p* = 0.013). Specifically, the allele T (mutant variant) was associated with greater reduction in positive symptoms scores that could be related with better response. Only one patient presented the T/T (Tyr452/Tyr452) genotype and there were 14 patients with C/T (His452/Tyr452) heterozygosity. Those with the C/T genotype showed lower values on the scales of psychotic symptomatology compared to the C/C (His452/His452) homozygotes patients (*p* = 0.051). No significant associations between the rest of the polymorphisms selected and scores on the applied scales were found.

### 3.2. Associations of Gene Polymorphisms Related with Pharmacokinetics and CLZ Response

CLZ plasma levels and NCLZ plasma levels were highly correlated (r = 0.764; *p* < 0.001).

A significant difference in pharmacokinetic parameters was associated with the polymorphism of *CYP1A2* gene encoding the main enzyme related to the metabolism of CLZ. Patient smokers (>7 cigarettes/day) with the *1F/*1F genotype for the *CYP1A2* gene had significant differences according to CLZ plasma levels and C/D CLZ (*p* = 0.029 and *p* = 0.034, respectively) versus *1/*1F and *1/*1 genotypes. Patients with *1F/*1F (n = 49) showed lower values with significant differences between the smoker (n = 20) and non-smoker (n = 29) population according to CLZ dose, C/D total, C/D CLZ, and C/D NCLZ (*p* = 0.002, *p* = 0.035, *p* = 0.046, and *p* = 0.032, respectively).

*CYP1A2* rs7625521 also demonstrated a significant association with the BNSS expression factor subscale (*p* = 0.007) between *1F and *1 allele. Patients with the *1F/*1F genotype were associated with a significant worsening of expression factor subscale (*p* = 0.037). None of the rest of the candidate polymorphisms of *ABCB1* demonstrated an association with pharmacokinetic parameters and the different scales.

## 4. Discussion

This study assessed the influence of pharmacogenetic polymorphisms on treatment response in TRS individuals. The CLZ response of the 100 participants in the present study was evaluated by GAF, PANSS, and BNSS scoring evaluation. Taq1A (rs1800497) polymorphism of *DRD2*, T102C (rs6313), and His452Tyr (rs6314) polymorphisms of *HTR2A* and 5-HTTLPR (rs4795541) and rs25531 of *SLC6A4* were analysed as relevant SNPs associated with pharmacodynamics of CLZ in TRS patients.

Our data suggest that patients who carry the T allele for rs6314 (*HTR2A*) might have relatively better CLZ response given that these patients presented increased improvement of the positive symptoms scores. Previous pharmacogenetics studies in populations of more than 100 Caucasian participants investigated the association between rs6314 SNP and CLZ response, suggesting that the T allele occurred more frequently in the non-responders group patients [26,27,28,40]. CLZ usually reduces psychosis in patients with schizophrenia because it shows a high affinity as antagonist or inverse agonist for the 5-HTR_2_ [19]. But recent findings suggest that CLZ may show agonistic properties in this receptor and was able to potentiate G-protein-depending signalling in cells expressing this receptor [41,42]. The rs6314 SNP of the gene encoding this receptor is an amino-acid substitution (His to Tyr) at position 452 that occurs in the intracellular carboxyl-terminal dominion of the receptor and may result in altered receptor structure and function [25]. A previous study found that the Tyr form (T allele) affects the signalling cascade of this receptor, such as G-protein activation and changes the response elicited by CLZ being less effective in terms of modification of phosphorylated peptides that may suggest a possible association with the poor CLZ response [43]. However, other reports did not show the same findings [44,45]. Malhotra et al. observed that BPRS scores after 10 weeks of clozapine administration had similar ratings for both genotypes, showing that the T/T genotype was non-associated with CLZ response [45]. Harvey et al. in an in vitro assay indicated that this SNP in the human 5-HT_2_A receptor does not significantly alter the response of the receptor to the antagonist CLZ [44].

Nevertheless, our findings do not show an association with allele C and a lack of CLZ response for rs6313 (*HTR2A*). Some studies reported that the TT genotype was associated with a better CLZ response in schizophrenia patients for the rs6313 [20,21]. The rest of results are controversial and many studies failed to replicate this finding [22,23].

Another polymorphism that was investigated for its association with clozapine response was 5-HTTLPR (rs4795541) of the *SLC6A4* gene. The *SLC6A4* gene is considered the most screened genetic variant in psychiatry. This polymorphism has been significantly associated with the CLZ treatment response, and it has been demonstrated that the S allele of 5-HTTLPR occurred more frequently in the non-responders’ group [21,30,46]. The S allele has a minor allele frequency of about 20% on a global average [47] and has been linked with decreased concentration of the transporter protein [48], and a poorer response to pharmacological treatment [49]. In the present study, the pharmacokinetic parameters versus polymorphism assessment showed an association between lower CLZ and NCLZ plasma levels and the presence of the S allele (mutant).

CLZ is metabolized primarily by CYP1A2, being the main route of CLZ metabolism. Previous studies showed that the *1F/*1F variant of rs762551 has been associated with non CLZ responders because of a reduction of the CLZ plasma levels [33]. It may therefore be hypothesized that a clinical response to CLZ treatment is related to the plasma levels of CLZ. Negative symptoms of schizophrenia manifest as reduced motivation and pleasure and impaired emotional expressivity [37]. In our study, this impaired emotional expressivity is more prominently observed in patients carrying the *1F/*1F genotype as compared to patients with at least one wild type allele for *CYP1A2* (*1 allele), potentially associated with a reduction in metabolism, leading to a poorer response. Furthermore, induction of this enzyme by smoking reduces CLZ levels and a lack of response has been seen in this population [8,50]. Our results corroborate these reports demonstrating that the patients who were smokers (more than 7 cigarettes/day) with this *1F/*1F variant showed a significantly lower CLZ C/D suggesting that tobacco induction may be greatly affected by the presence of this genotype.

Managing UTRS poses ongoing challenges within the psychiatric field. Typically, the primary approach entails enhancing treatment plans by incorporating different medications such as additional antipsychotics. However, despite these endeavours, there exists limited evidence supporting the efficacy of these augmentation strategies [51].

In our study, we observed that patients requiring augmentation therapy exhibited poorer symptom outcomes despite having higher doses and plasma levels within the therapeutic range. Possibly, with these medications, the patient’s therapeutic response may be improved, but it remains lower than in patients treated only with CLZ, which implies a worse therapeutic response. However, in terms of genetic predictors of CLZ response, our results show no significant differences between both groups. This may be because it is unlikely that the response to CLZ is dictated by a single genetic variable and could be due to the association of multiple SNPs.

We are aware that our study has several limitations. In the case of a genetic association study, the relatively small sample was the most important limitation. Therefore, it is necessary to validate our results in a larger cohort of patients. Even though there are studies exploring the influence of other genes on the outcomes of CLZ pharmacotherapy, only those we deemed to have more validity in previous studies were selected. The response classification is complex; the differences in the associations between genotypes or alleles and the different response groups (phenotypes) could be related to the heterogeneity of definitions or criteria. The classification of a patient as responder or not differs depending on the studies and each psychiatric institution that makes it difficult to compare certain results. In the reviewed previous studies, significant variability is observed in the criteria used to determine response or non-response to CLZ based on different psychotic scales (PANSS, GAF, or BPRS). Additionally, the duration of CLZ treatment must be considered. It is indicated that most patients will have a higher percentage of response after 12 weeks of being on CLZ treatment, so studies with patients undergoing CLZ treatment for a shorter duration may be too short to establish treatment response [52].

To date, the specific mechanisms underlying the efficacy of CLZ in patients with TRS and the definitive biomarkers of response to CLZ have not been clearly identified. Although the role of pharmacogenetic testing had some significance in our study for certain SNPs, the availability of testing in psychiatric clinics remains limited. Overall, despite mixed results and a limited number of robust genetic predictors, CLZ pharmacogenetics continues to hold potential for improving treatment in patients with schizophrenia [53]. Considering clinical and demographic confounding factors in pharmacogenetic studies may help achieve greater consistency in research studies, leading to an improvement of response prediction.

Therefore, the future of CLZ pharmacogenetics, through the incorporation of genetic testing for genes involved in its mechanism of action and metabolism, holds great potential for clinical implementation. This provides psychiatrists with the opportunity to utilize this drug at its maximum potential, tailored to each patient, and likely reduce resistance to CLZ by accurately identifying its cause. More studies of this type are needed to increase the scientific evidence and the real utility of this science when applied to routine clinical practice.

## 5. Conclusions

In conclusion, we can indicate that the response to treatment with CLZ in patients with TRS reveals certain significant findings. Patients who exclusively responded to CLZ exhibited distinctive features, such as lower CLZ doses, lower plasma levels, and less pronounced symptomatology.

Additionally, the study explored genetic associations, mainly focusing on polymorphisms associated with CLZ pharmacodynamics in relation to treatment response. Genetic studies related to pharmacodynamics are challenging to interpret due to the complexity of the CLZ mechanism of action. The S allele carriers of *SLC6A4* gene showed the lowest CLZ and NCLZ plasma levels versus L allele carriers. However, at the pharmacokinetic level, we observed that the *1/*1F polymorphism of *CYP1A2* showed associations with CLZ plasma levels, especially in smokers; we propose that this genotype may be associated with a lack of response (aligning with negative symptoms) in psychotic patients. This suggests that genetic variables associated with pharmacokinetics may have a greater impact on CLZ response.

Nevertheless, it is crucial to consider the study’s limitations, such as sample size and the complexity in classifying treatment response, marked by variability in criteria and the duration of CLZ treatment. All of this highlights the need for more standardized approaches in future research to enhance understanding of the biomarkers influencing CLZ efficacy.

## Figures and Tables

**Table 1:** Summary of gene polymorphisms which might impact CLZ treatment.

Gene	Variant	Genotype/Allele	Predicted Phenotype	Reference
*DRD2*	rs1800497 (Taq1A)	A1/A1 or T/T *A1 allele or T allele	Better response CLZ	[18]
*HTR2A*	rs6313 (T102C)	C/C C allele	Non-response CLZ	[20,21]
*HTR2A*	rs6314 (C1354T or His452Tyr)	T/T T allele or 452Tyr	Non-response CLZ	[26,27,28]
*SLC6A4*	rs4795541	S/S or S allele	Non-response CLZ	[29]
*SLC6A4*	rs25531	G/G or G allele	Non-response CLZ	[29]
*CYP1A2*	rs7625521	*1F/*1F or A/A A or *1F allele	Low CLZ plasma and non-response	[33]
*ABCB1*	rs1045642 (C3435T)	T/T or T allele	High CLZ plasma and better response	[34]
*ABCB1*	rs2032582 (G2677T)	T/T or T allele	High CLZ plasma and better response	[34]

**Table 2:** Sociodemographic and clinical variables of the 100 patients included in the study.

Variables	Number [%] or Mean (S.D)
Age	50.26 (11.11)
Gender (=male)	86 [86]
Smoking (Cigarettes/day)	6.49 (10.50)
Duration of CLZ treatment (years)	15.09 (8.12)
CLZ dose (mg/day)	337.18 (153.69)
CLZ plasma levels (ng/mL)	419.4 (187.08)
NCLZ plasma levels (ng/mL)	253.1 (104.49)
Ratio CLZ/NCLZ	1.69 (0.46)
C/D CLZ	1.422 (0.77)
C/D NCLZ	0.845 (0.41)
C/D total	2.267 (1.13)
Wellbeing (SWEMWBS) score	23.89 (4.66)
Genal functioning (GAF) score	73.18 (11.55)
PANSS-positive	13.26 (4.50)
BNSS overall score	23.00 (15.42)
BNSS Motivation and Pleasure Factor	14.44 (9.27)
BNSS Expression factor	8.57 (7.53)

**Table 3:** Clinical characteristics between patients who respond to only CLZ (CLZ-TRS) and those requiring augmentation strategies (CLZ-UTRS).

Variables	CLZ Only (CLZ-TRS) n = 49 Mean (S.D)	CLZ + Augmentation Strategies (CLZ-UTRS) n = 51 Mean (S.D)	*p* Value
CLZ dose (mg/day)	306.75 (167.87)	365.13 (135.16)	0.033 *
CLZ plasma levels (ng/mL)	354.6 (187.01)	476.6 (169.14)	<0.001 **
NCLZ plasma levels (ng/mL)	217.0 (118.07)	284.9 (79.21)	<0.001 **
Wellbeing (SWEMWBS) score	25.21 (4.90)	22.62 (4.07)	0.003 *
Genal functioning (GAF) score	74.46 (13.14)	71.96 (9.76)	0.141
PANSS-positive	11.58 (3.96)	14.93 (4.42)	<0.001 **
BNSS overall score	20.62 (15.54)	25.39 (15.09)	0.072
BNSS Motivation and Pleasure Factor	13.47 (9.10)	15.42 (9.44)	0.160
BNSS Expression factor	7.15 (7.48)	9.98 (7.38)	0.037 *

* *p* < 0.05. ** *p* < 0.001.

## Data Availability

The research data can be requested from the first authors (E.S. and E.F.-E.) and from the corresponding author (C.B.G.). Due to privacy and ethical restrictions, the data underlying this study cannot be made publicly available.
